# Viral Ancestors of Antiviral Systems

**DOI:** 10.3390/v3101933

**Published:** 2011-10-20

**Authors:** Luis P. Villarreal

**Affiliations:** 1 Center for Virus Research, University of California, Irvine, CA 92697, USA; E-Mail: lpvillar@uci.edu; Tel.: +1-949-824-6074; 2 Department of Molecular Biology and Biochemistry, School of Biological Sciences, University of California, Irvine, CA 92697, USA

**Keywords:** virus evolution, prophage, evolution of immunity, restriction modification, addiction modules, toxin antitoxin, apoptosis, CRISPR, RNAi, interferon, adaptive immunity, T-cell receptor, MHC, ERV, HERV, LTR

## Abstract

All life must survive their corresponding viruses. Thus antiviral systems are essential in all living organisms. Remnants of virus derived information are also found in all life forms but have historically been considered mostly as junk DNA. However, such virus derived information can strongly affect host susceptibility to viruses. In this review, I evaluate the role viruses have had in the origin and evolution of host antiviral systems. From Archaea through bacteria and from simple to complex eukaryotes I trace the viral components that became essential elements of antiviral immunity. I conclude with a reexamination of the ‘Big Bang’ theory for the emergence of the adaptive immune system in vertebrates by horizontal transfer and note how viruses could have and did provide crucial and coordinated features.

## 1. Introduction

Metagenomic studies inform us that viruses are the most numerous and diverse genetic agents in essentially every habitat that has been examined [1–10]. Comparative genomics also informs us that virus derived information is abundant and lineage specific in all life forms. Although such viral information is often defective and historically considered junk, it is also an excellent record of the paleontology of genomes. As genetic parasites, viruses must be compatible with yet extend the existing genetic code of the host. This seemingly simple feature has deep implications and it has been proposed that viruses are inherent and essential editors of host genetic code [11,12]. Wherever we find life we also find abundant genomic or extragenomic viruses. However, awareness of this virus-host ecology was not part of original Darwinian theory. The most common view is that viruses are harmful genetic parasites that can often kill their host. This well accepted and supported paradigm strongly colors our perceptions of how viruses and host interact and evolve. Viruses are thus modeled as predators to their prey host. Lytic virus destruction of host, sets up an arms race in which clonal selection of host variant (often virus receptors) results in virus resistant new host population [13]. Viruses can then adapt to new populations to ‘kill the winner’. This is classical Darwinian selection of the fittest individual type based on host variation and natural selection. Such a process should result in a slow and stepwise accumulation of genes that resist viruses derived from ancestral genes that can provide host immunity. This process also would not need to invoke any ‘big bang’ or complex horizontal transfer event to create virus immunity. However, even the earliest virus-host studies proposed virus-induced virus resistance [14], and others observed that host virus resistance could also be mediated by other virus [15], even defective ones [16]. A long standing problem with understanding the origin of host virus resistance is explaining how the host wins this battle. Since viruses replicate exponentially, can tolerate even lethal errors at high rates and can freely exchange information with host and other virus, they would seem to always have the upper hand in evolution. Indeed they have been measured to evolve up to a million times faster then the host [17]. Given this situation, how can the host build defense network or system against even yet unseen virus? But viruses are not simply run-away replicators. They are also tenacious colonizers of host able to resist and preclude competition. In this review, I outline the evidence and arguments that viruses themselves often provide the host with antiviral defenses.

Early in my introduction to virology, I became interested in cryptic virus-host states that differed from the classical lytic or pathogenic state. Persistence of virus information seemed to be broadly seen in most life forms, generally with no pathology. Persistent relationships are now known to be ubiquitous, stable and highly host specific [18]. They define a form of virus-host living together that fits within the definition of symbiosis [19]. However, symbiotic does not equate to mutualistic which leads us to ask if these viruses can provide good outcomes to their host. Indeed, examples of beneficial symbiotic viruses are now starting to be acknowledged [20]. However, one aspect of this situation that has received less attention is whether symbiotic viruses have consequences to the same or other viruses. Is there a virus-virus dynamic (such as antiviral immunity) that extends beyond virus-host dynamic? Do these cryptic and often endogenous viruses have consequence to the viral immunity of their host? In some cases (for example, see [21]), the answer is clearly yes, but the generality of this question remains unanswered. Below, I compile evidence for the general role of viruses in the origin of antiviral immunity.

## 2. Prokaryotes

Early in the study of lytic viruses of bacteria, it was observed that host virus resistance could be due to the presence of a second persisting and often genomic virus that would oppose the lytic virus. For example, various common *E. coli* lab strains were discovered to harbor P1 (an episomal temperate phage initially considered a plasmid) that would render the cells resistant to the iconic T4 and lambda phage infections. And even defective (cryptic) prophage (resembling junk DNA) of various types (such as lambda) could affect host susceptibility to various lytic viruses such as T4. These resident viral agents were also capable of horizontal transfer (infection, colonization), thus quickly imparting other host with antiviral states. Thus, prophages can clearly provide an immune function to host. For example, the iconic *E. coli* K-12 harbors an e14 defective lambdoid cryptic prophage that encodes T4 exclusion, and cytosine restriction functions [22]. Indeed, the long studied but poorly understood iconic rexB gene of lambda appears to be an anti-death gene most likely associated with phage exclusion [23]. In addition, other viruses can mediated the phenomena of superinfection exclusion in which a temperate phage efficiently prevents further infection with the same or related virus, see [24]. And some viruses (e.g., P2) can more generally inhibit other viruses (e.g., T-even) [25]. Like viruses, plasmids also provide antiviral systems. Plasmid exclusion is a well known phenomena, see [26]. Historically, viruses and plasmids have been considered as distinct. However, in many cases the mosaic nature of gene exchange between them increasingly makes these distinctions vague, see [27–31]. The P1 example also blurs the distinction between virus and plasmids. However, it was with P1 that a role for T/A (toxin antitoxin) gene sets in inhibiting second infectious agents was first clarified. P1 is an episomal phage that was originally also thought to be a plasmid, see [32]. However, P1 opposes both its loss from host populations and infection with other viruses by the action of programmed cell death (via T/A disruption) [33]. Indeed, the existence of programmed cell death for clonal organisms like bacteria seemed antithetical. One of the gene pairs that P1 uses as a T/A gene set is a restriction-modification (R/M) set. Since R/Ms are major antiviral systems of prokaryotes, the viral role in R/M evolution is considered below. We know of numerous processes involving virus/plasmid by which prokaryotes become resistant to virus [34]. We also now know that most of the genetic variation in the initially sequenced *E. coli* genomes could be ascribed to the action of phage [35]. Comparative genomics makes clear that the dominant process of bacterial and Archaea evolution is through horizontal gene transfer involving gene sets [36]. Viruses are clearly major contributors to this mosaic acquisition process [37] as integration to adjacent tRNA sites is mostly phage associated [38]. Interestingly, there seem to be general differences in phage–plasmid interactions between bacteria and Archaea [39], suggesting domain wide distinctions in their virus and antivirus relationships.

## 3. The R/M System, T/As and Virus

As mentioned above, R/M genes can also be part of a maintenance and apoptosis system in which self destruction is induced with loss from the bacteria [33]. Thus R/Ms can be considered a T/A gene set (genotoxic R, protective M) that induce post segregation self killing [40]. This gene set can also be called an addiction module. Similarly, Eco R1 in *E. coli* is also involved in post segregation killing (as an addiction module). Type II R/Ms are the most common, diverse and a prokaryote-wide system of phage defense genes [41]. Since restriction enzymes recognize small palindrome sequences, host genomes are under R/M mediated phage selection and have shown clear biases regarding the frequency of these sequences in all prokaryotes [42]. R/M gene sets seem especially prevalent in the colony forming filamentous cyanobacteria [43] whose sessile and colonial life strategy may need to resist many viruses [40]. Clearly, phage can encode R/M sets as a T/A set and these have the features of an addiction module. Other phage are also known to encode T/A gene sets [44]. R/M is also found in plasmids, but here they are often also flanked by phage derived sequences [45]. In E. coli, horizontal transfer or variation in R/M systems can be mediated by plasmids that are clearly P1 like [46]. Thus, in many cases, R/M horizontal transfer has occurred with phage or phage assistance. Furthermore, codon word bias confirms that R/M systems (and prophage genes) are often of ‘alien’ origin and acquired by horizontal means [47]. In some bacteria (*Haemophilus influenzae*), phase variation exists in repeat tracks expressing the R/M system along with the lipooligosaccharide phage receptor promoter to generate more diverse phage resistance [48]. This represents a more elaborate but still R/M derived defense against phage. Thus, the R/M system, the most diverse prokaryotic antiviral system known, shows strong evidence that viruses are crucial for their origin and evolution and are guarding the host genome.

## 4. Toxins, Host Adaptation and Phage

Our focus is on the origin of antiviral systems. Yet, ironically, virus (e.g., provirus) can be crucial for bacterial adaptability, thus a fully effective antiviral system could limit host adaptability. This is particularly apparent regarding the link between bacterial prophage and toxins, including defensive R/M genes, see [49]. Toxin and R/M genes are crucial for certain habitat adaptations and such habitat adaptation is also associated with virus defense genes. Toxins are particularly abundant in marine bacteria [50]. Similar bacteria from distinct locations (*Candidatus Accumulibacter phosphatis*) will differ predominantly in regions encoding phage defense genes [51]. Prophages also contribute to habitat adaptation outside of R/M genes. In one example of deep sea hydrothermal vents, metagenomic analysis indicates that inducible temperate prophages are common and contain surprisingly numerous novel genes [52]. In other cases, it seems clear that bacterial phage are not simply promoting clonal sweeps as they appear to maintain host diversity [53]. The link between adaptability, toxin and phage has especially been studied with toxigenic strains of *E. coli* (O157) that differ in toxin carrying cryptic phage sets (especially lambdoid) [54–59]. These horizontally acquired genes are transferred via the promoting activities of defective and cooperating prophages [60], which can also provide globally coordinated gene regulation [61]. Even commensal *E. coli* strains harbor similar but strain specific cryptic phage [62]. These observations are consistent with a general role of lysogenic prophages in pathogenicity (and adaptability) of bacteria as has been previously proposed [63]. There is also a relationship between cryptic phage/plasmids and biofilm formation, another process of bacterial adaptability. Elimination of cryptic prophages from *E. coli* (including their encoded T/As) results in lost biofilm formation [64,65]. Abortive virus infection can also be toxin mediated. Diverse bacteria have T/A gene pairs, like R/M, that can preclude or abort infections with diverse phage via toxin mediated self destruction [44,66]. One prevalent version of this abortion system uses tandemly repeated Toxi RNA to counteract ToxN gene function [67]. Indeed, pathogenic island transfer between bacterial species has also been observed mediated by phage [68]. Furthermore, T/A gene sets also seem involved in persister cell formation [69], which can also affect virus production. Thus, phage derived ‘antiphage T/A defense systems’ often provide the host not only with adaptations against other viruses, but also with adaptations for better habitat colonization.

## 5. CRISPRS

CRISPRS are clusters of regularly interspaced palindromic repeat DNA sequences linked to CAS genes that are found in both Archaea and bacteria but more prevalent in Archaea. The locus provides RNA directed immunity against viruses and plasmids, see [70,71]. The process involves dsDNA recognition via guide CRISPR RNA (crRNA) [72]. Some CRISPR loci can also counteract plasmids via self splicing intron insertion into plasmid DNA [73]. CRISPR is an effective but mostly antiviral and anti-plasmid system that retains memory of past viruses by incorporating identity-recognition relevant information from these viruses [74]. This memory thus provides a powerful and advanced adaptive feature. Indeed, the variable spacer regions of CRISPER loci has been used to detect previously unknown viruses that have infected the host [75]. Memory is thus a distinct feature of CRISPR. In Archaea, the majority of virus infections are chronic, although some are also clearly lytic [7]. These archaeal viruses have numerous distinct morphotypes, which are thought to represent ancient lineages [76]. Some archael plasmids can also be packaged and spread by virus [77]. In contrast to bacteria, however, Archaea appear to lack RNA viruses. It is not yet known if CRISPR loci can account for this difference. CRISPRS (like T/As) can also be needed for group (biofilm) behavior [78]. Since the distribution of the CRISPR palindromic repeats in virus/plasmid genomes is random, the prevailing hypothesis is that these repeats are derived from these viruses and plasmids [79]. The CRISPR loci shows uneven coevolution with their host. In general, CRISPRS are species conserved and stable during host evolution. However in *E. coli*, the corresponding CAS gene is rapidly changing and incongruent with CRISPR loci and host species, thus it has a distinct and horizontal evolutionary history [80]. This observation indicates the CRISPR role is not simply to protect bacteria from phage, but likely has additional functions, such as a possible role in community structure. However, some have proposed that an effective CRISPR system may limit prophage colonization (especially in Archaea). It is thus interesting that Archaea and bacteria also differ considerably with respect to toxin production in general, given that toxins are most often prophage encoded [81].

## 6. Eukaryotes Shift Virus and Antivirus Defenses

Interestingly, R/M systems are almost entirely absent from eukaryotes, although they are well conserved in the chloroviruses (PBCV1), large DNA viruses that infect unicellular eukaryotic green algae [82]. In addition, type II restriction-like genes (R lacking M) are reported in R4 family retrotransposons found in invertebrate and vertebrate [83]. The prokaryotic wide R/M defense systems are thus clearly no longer significant components of eukaryotic antiviral defense. Nor is the CRISPRS system retained in eukaryotes as neither the repeat loci nor the CAS gene is found therein. Given the major viral role in bacterial adaptability discussed above, why might this relationship have changed so completely with the emergence of eukaryotes? Along with this global change in antivirus defense, the integration of large ds DNA viruses into host chromosomes (*i.e.*, prophage life strategy) became most uncommon in eukaryotes. Instead, we see a large scale increase in the integration activity of retroviruses and retroposons into eukaryotic chromosomes. And although the CRISPR system was not retained, we instead observe the emergence of an RNA based antivirus defense system that resembles CRISPRS in many regards (such as dsRNA directed virus recognition). Below I consider the likely role of viruses in the emergence of eukaryotic antiviral systems.

## 7. *C. elegans*, RNAi and Antivirus Systems

The model nematode worm *C. elegans* has been productively studied regarding RNA based antivirus systems. Like all other eukaryotes, *C. elegans* lacks the CRISPRS loci, R/M systems, DNA prophage, or plasmid exclusion systems as found in prokaryotes. But *C. elegans* does have an effective RNA based antiviral response. The process operates via small or micro RNAs; small ssRNA that interfere (RNAi) with gene expression [84] or silences (siRNA) gene expression [85]. Components of this system are conserved in most, but not all, eukaryotes. The small RNA response can sometimes affect genes in groups [86]. Some of these small RNAs can be expressed from genomic DNA (corresponding to LTR regions of endogenous retroviruses and transposons, presented below). This system can provide antiviral state via virus derived siRNA [87]. The origins of this system have not been defined and no prokaryotic homologues are found. The system involves an RNA dependent RNA polymerase (RDRP) to amplify the signal RNA [88]. As dsRNA based genetics is only in the domain of viruses (not host) this clearly resembles a virus-like strategy. This gene has no counterparts in the Archaea or bacteria [89]. Nor has a cellular ancestor to this RNA pol been identified. However, the RNA pol does show some domain similarity to a phage DNA dependent DNA pol, thus some type of viral mosaic origin seems possible [89]. In addition, filamentous fungi (considered basal to metazoans) are typically chronically infected with diverse dsRNA viruses that encode related genes and these genes can be truncated and/or acquired by horizontal gene transfer [90] (see below). Another component of this system involves the cleavage of dsRNA via the action of RNase III (Dicer PAZ domain). Like RDRP, RNAse III also lacks homologues in Archaea and bacteria [90] and appears to have been acquired by horizontal transfer. Adding to this evidence of virus-like features is the highly transmissive nature of the RNAi response in *C. elegans* [91–93]. Such amplifying cell to cell transmission clearly resembles a virus-like strategy, further suggesting viral origins. However, all these observations are only inferential and more direct evidence is needed.

Nematodes seem to have a very effective antivirus system. Given the large and old collections of cultured nematodes, especially with respect to their roles as plant disease vectors, and given the length of the use of *C. elegans* as a laboratory model, there is a surprising paucity of reports of viruses that infect nematodes. Recently, persisting non-pathogenic infections with noda-like [94] and rhadbo-like viruses have been reported for nematodes [95]. Clearly, nematodes have yet to show the large virus mediated population crashes we describe below for marine vertebrates and invertebrates or are well established for bacteria. Their effective antiviral system is based on dsRNA response that targets RNA via small RNAs [96]. And if incapacitated, this system renders the worm susceptible to pathogenic infection with various RNA viruses [87,97–100]. A main component is a dsRNA binding protein and an endonuclease to degrade the corresponding mRNA into siRNAs. Indeed, dsRNA recognition via small RNAs (such as RNAi, siRNA, microRNA or miRNA) is a general theme of antivirus immunity not restricted to invertebrates that we can follow to plants and vertebrates [96]. Unlike the R/M system described above, however, it does not generally appear to act as a T/A system.

In *C. elegans* the majority of small RNAs are endogenously expressed, mostly deriving from endogenous LTR transcription. Indeed, the *C. elegans* genome underwent a substantial colonization by Gypsy-like ERVs (chromovirus) during its evolution and although most are defective some are essentially intact [101]. The LTRs from these retroviruses were scattered throughout the genome and this has led to proposals that dsRNA response was initially a defense against retroviral elements [102,103]. If, however, the ERV colonization was itself important for the origin of this antivirus system, we can instead assert that this dsRNA system was derived from retro and other viruses to preclude self and other viral competitors (by providing self identity and allowing stable ERV maintenance) [104]. Thus, this could explain why it appears to be a defense against other viruses yet account for why LTRs remain responsible for the expression of endogenous small RNAs. This system is thus guarding the genome against non-self elements while allowing coexistence with ‘self’ ERVs [105,106]. siRNA was clearly conserved in other more complex invertebrates, but here the small RNA may more often be derived from exogenous viral, not LTR RNAs. Accordingly, it may be possible to use siRNA to identify previously unknown exogenous viral genomes that have infected or are colonizing their host. This idea is very much like that applied to the CRISPRs system noted above [107]. Interestingly, although this approach was effectively used in *Drosophila* (ovary) to find new viruses [108], in *C. elegans* no viral genomes were thus identified. This suggests that there are indeed few persisting viruses in *C. elegans*, consistent with a virus paucity discussed above. Curiously, in *Drosophila*, piwi interacting RNA is also thought to be an anti-transposon RNA (via flamenco rasi RNA, antisense to gypsy elements) [108], but piwi RNAs are expressed only in the ovary [109], as are gypsy ERVs in some fly strains. This is a curious overlap of ERV and anti-ERV putative function. However, all fly strains retain gypsy defective elements. Indeed, one-quarter of all cloned small RNAs from *Drosophila* are also derived from such retro elements [110]. Together, these observations can support the assertion that retroviruses are indeed involved in the origin of these antiviral systems.

## 8. Paleovirology: The Filamentous Fungi

Like other early metazoans, fungi have most of the small RNA genes (including dicer) noted above, but the specific occurrence and composition of such genes is more diverse in the early metazoans [111,112]. The viral susceptibilities of filamentous fungi is also distinct from those of both prokaryotes and simple animals (*C. elegans*). Many RNA viruses are known to infect fungi, but especially interesting is the ubiquitous persistent and nonpathogenic colonization by various dsRNA viruses [113,114]. These silent, cryptic viruses [115] mostly have no extracellular mode of transmission and depend on fusion of hypha for transmission [116,117]. Indeed, some have proposed that the existing hyphal mating type restriction is for the purpose of virus control. Furthermore, some of these viruses can provide selective and pathogenic (toxin) phenotypes to their host fungi [118], whose transmission is also mating type restricted [20,115,119,120]. A core RNA viral gene is the RDRP. Recently, reports of widespread horizontal ‘module’ transfer of dsRNA viruses (especially RDRP) into the nuclear genomes of eukaryotes have been published [121]. Such ubiquitous infection of filamentous fungi with dsRNA viruses clearly distinguishes the situation form that seen in *C. elegans* as noted above. Consistent with this inference, the conservation of the antiviral small RNA genes is also most uneven in fungi. These genes have been conserved and multiplied in some lineages but are also truncated in various other filamentous and nonfilamentous fungi [111,122]. It seems likely that the presence of these antiviral systems will also affect virus colonization. Of great interest with respect to likely origins is that some filamentous fungi do not use cellular RDRP for inverted repeat RNA silencing [123]. Fungi also seem to have some distinct recognition systems not seen in other species. For example, the RIP system of neurospora precludes the accumulation of genomic (non-nucleoli and non-plastid) repeats by heavy mutation of LTRs and ERVS during sexual reproduction [124,125]. Yet other lineages of fungi have significant LTR content which is associated with pathogenic adaptation [126]. The fungal ERVs are especially of chromoviral origin [127]. Thus in the fungi, viral specific associations and involvement in antiviral systems seems basic to their evolution.

## 9. Paucity of Tunicate Virus, Antivirus States and the Problem of Gradualism

It is a long evolutionary jump from worms to people if we think about the continuing emergence of immune systems. Our interest thus turns to the tunicates, as the most likely ancestor to living vertebrates [128]. Tunicates have a highly polymorphic fusion/histocompatibility locus (FuHC) associated colony fusion or destruction [129,130]. In addition, lampreys and hagfish have distinct, highly diverse and modular variable lymphocyte receptors (VLRs) that promote lymphocyte pattern recognition [131,132]. These loci are both considered to also provide pathogen immunity. Neither of these loci bear similarity to classic genes of the adaptive immune system of jawed vertebrates. Nor do they have the classic interferon alpha (dsRNA response) or interferon gamma system, although they do have complement [133] and Thy-1 like genes [134,135]. It is clear that these tunicate systems emulate various features of the adaptive immune system, even though they lack specific homology [136,137]. Thus their emergence was clearly a distinct evolutionary event. Chromoviruses (gypsy) remain abundant and active in tunicate genomes [138,139]. Yet tunicates retain the smallest of all vertebrate genomes [139,140]. Currently, and most curiously, there is no evidence that these tunicate systems function as antiviral systems. Unlike the small RNA system of *C. elegans* presented above, the antiviral consequences of mutations in the tunicates systems have not been evaluated. This may partially be due to the paucity of viruses so far found in tunicates. There are no laboratory models for tunicate infection with virus, possibly indicating that tunicates have effective antiviral systems. One recent report using metagenomic approaches did find the presence of rhabdovirus RNA (VHSV) in the European river lamprey [141]. However, this appeared to be a cryptic, nonpathogenic infection with unknown linkage to the VLR system. Thus tunicates resemble nematodes in the low occurrence of natural virus susceptibility. Although we cannot link these tunicate systems to that of jawed vertebrates, many researchers are still proposing a gradual transition to the origin of adaptive immunity via convergent evolution [137,142]. One of their main criticisms against the long held ‘big bang’ theory is that the acquisition of all the complex linked features of adaptive immunity is considered too ‘extreme’ [142,143]. Gradual evolutionary mechanisms are instead favored. However, arguments that acknowledge the evidence for or major effects of horizontal transfer do not appear to underlie these views. Rather they appear to be based solely on the belief that only point mutation and natural selection is needed to account for such genetic novelty, thus it is posited that whole genome duplication and slow adaptation of individual genes co-opted from various other uses can fully explain the origin of adaptive immunity [142–143]. That no living organism resembles the intermediate required for this gradualist process is apparently not a major concern. More fundamentally, however, I disagree with these views because they fully ignore a much deeper issue of the regulatory complexity and network coherence that is required for originating the adaptive immune system. The adaptive immune system is an especially troubling example of a novel complex regulatory network of interacting genes that was absent from any living ancestor. Even ignoring all the needed new genes, simply solving the dispersed editing and regulatory requirements of adaptive immunity presents a major conundrum by itself. This indeed requires a ‘big bang’ like event of complex horizontal colonization by a population of linked agents with coherent regulatory competence. Indeed, a most basic problem regarding editing of the genetic code in general is that needed to create a new network. The fundamental hurdles to coordinate distributed code to the degree needed to generate such regulatory complexity are much more challenging and difficult then has previously been acknowledged [144,145]. Indeed, this problem relates directly to the nature of code ‘meaning’ itself since edited preexisting code (*i.e.*, co-opted genes and regulators) must be assigned new meaning in the context of the new network. An incremental and individual based process for this is not sufficient. Point mutation errors followed by selection of the fittest type cannot account for such network coherence. However, the colonization by a population of linked agents can promote coherence or network formation [146]. I propose here that mosaic populations of mixed viruses and their regulatory elements are naturally competent in host code usage (especially that used by immune systems) and are also natural extenders (editors) of new (virus derived) code. Let us now examine the origin of the adaptive immune system from this perspective.

## 10. Components of Adaptive Immunity of Jawed Vertebrates: Big Bang Reexamined

The early ‘big bang’ view of the origin of adaptive immunity required the horizontal transfer into emerging vertebrates of almost the entire system *en toto*. This system could then evolve into greater complexity as seen from fish to humans [147]. But where did such a complex system come from if not from an ancestral organism? In a prior publication, I presented a detailed and in depth proposal of how and why viruses and other genetic parasites would provide the likely origin of the adaptive immune system [148]. As none of the recent ‘gradualist’ reviews on the adaptive immune system have addressed or critiqued the main assertions made therein, the evidence and arguments previously presented remain pertinent. Below I outline those main issues and provide some crucial references that relate to the role viruses and genetic parasites played in the origin and evolution of adaptive immunity. Teleost fish genomes underwent a big virus colonization (ERV) and genome expansion relative to tunicates [149]. This involved a significant shift including both chromoviruses expansion (Gypsy-like ERV) and colonization by many other new retrovirus families [150,151]. Along with this major genome change, we see that vertebrates acquired all of the core elements of adaptive immunity. This also included a major shift in innate immunity (including altered apoptosis and interferons—alpha and gamma). The specific elements of adaptive immunity include the antigen specific cytotoxic T-cell (CTL), antigen processing and presentation (via MHC locus), adaptive antibody, memory, and IFN alpha and core regulation by IFN gamma [152,153]. The organs and tissues needed to produce all this also emerged with the vertebrates. This system functions as a complicated and coherent network. However, the regulatory IFN system of fish also shows direct antiviral activity (not antigen specific) [152]. Vertebrates also show a big increase in the complexity of their apoptotic response [110]. It has been argued that most of these changes appear to have originated via horizontal gene transfer.

It is commonly assumed that the adaptive immune system with its anticipatory capacity is much more advanced and capable then the ancestral immune system. We have seen, however, that effective antiviral systems based on RNA were widely present in various invertebrate ancestors in highly virus infested habitats. In contrast to tunicates, fish have lots of viral disease. This includes a diverse set of viruses that can cause large population crashes in both natural and farmed fish populations. For example, teleost fish population crashes due to rhabdoviruses, herpesviruses and infectious retroviruses that transform immune cells have all been frequently observed [154,155]. Indeed, viral infection of immune cells is rather common in the jawed vertebrates. So was the adaptive immune system better for controlling viruses? Possibly not. Nor is there evidence for large selective sweeps by viral pathogens of ancestor populations in the geological record. Thus it is not clear what selective pressure led to the emergence of adaptive immunity. One interesting idea concerns the invention of teeth and jaws used by predators in the consumption of living prey (mostly marine invertebrates). The resulting exposure to wounds, blood and ubiquitous virus from marine invertebrates could have promoted the origin of adaptive immunity [156]. Another view is that an ancient form of antiviral immunity led to the adaptive immune system [157]. Let us now follow this perspective and consider how it might explain the various extant features of adaptive immunity. These central features are conserved from fish to humans (who also have the most complex MHC locus) and are outlined below [147]. Along these lines, cytotoxic T-cells (CTLs) would have initially emerged to kill virus infected cells. The T-cell receptor is found within a 1 Mbp cluster of interacting genes (via protein-protein interaction), which formed contemporaneously with the origin of the adaptive immune system [158]. These did not originate by genome duplication, nor are they found in chordates. Of particular interest is that the MHC locus is the most dynamic and gene dense region in the chromosomes of fish and humans. However, it made dynamic via the action of ERV and other retroposons as described below.

## 11. The T-Cell Receptor (TCR and Ig Family)

It has been pointed out that a cellular virus receptor will bind its virus with good affinity, but if the receptor is also released into the media, it can potentially also bind and inactivate free virus, thus providing the basis for soluble antibody to bind virus (Ig) [159]. The TCR appears to be the most basal form of the Ig family members of the immune system. However, even more ancestral features of the Ig superfamily are found in various virus receptors, such as CTX (Coxackie receptor), PRV (poliovirus receptor), JAM (ds RNA reovirus). Similar receptors can be found in Ciona. Thus it has been proposed that these virus-binding properties were recruited for origin Ig superfamily in the adaptive immunity, then secreted to produce early virus neutralizing activity [159,160]. Along these lines, the adenovirus CAR receptor is known to be shed as a soluble form [161]. However, as outlined in my paper, and noted briefly above, viruses themselves are often the source of the most variable virus receptors [148]. In addition, there are several examples of virus receptor systems that also encode a linked system for generating variation in the receptor. The colonization of the host genome by such a virus that encoded a variable receptor system would provide the foundation TCR (and Ig) variation. Along these lines, a very interesting situation is found in sharks (basal vertebrates with adaptive immunity). Sharks express high levels of natural antibody (from unrearranged Ig genes) [162]. These antibodies have affinity to TCR but also for numerous retroviral proteins, especially envelope proteins. This natural affinity may identify the original target of the Ig immune proteins and would be consistent with a very early retroviral involvement.

## 12. The TCR ‘Receptor’ Must Link to a Recombination System

The recombination and variation of TCR and the Ig family members occurs via the site specific action of RAG1/2 recombinase involving sequence specific binding for V(D)J recombination. These two genes are not found in tunicates or most other invertebrates [163]. It has long been noted that this integrase has features of phage and viral integrases (especially that of phage Mu needed for replication). As RAG1/2 also targets small repeat sequences for integration, several early proposals suggested viral involvement in the horizontal acquisition of RAG1/2 [164,165]. Clearly the RAG1/2 recombinase along with its target sequences resembles an ancient site specific transposable element. More current views, however, propose that RAG1 and RAG2 were derived from selfish DNA transposons via horizontal transfer [166]. However, how this recombinase became regulated and linked to the TCR genes or how this might have occurred without involving viruses is mysterious. It is typically assumed that classical Darwinian selection was sufficient to provide this needed linkage and coordination. In my prior review, however, I provide a detailed scenario of how a ‘preevolved’ virus based system for receptor gene variation with inherent links to an integration process could colonize the genome and provide the foundation of this needed TCR-RAG link [148]. Numerous and highly conserved examples of homing introns found in specific viral core genes are known. Similar systems for viral receptor gene variation are also known. Thus host colonization of mixed viral receptor module with a coordinated variation system would allow the acquisition of a coherent system much more quickly, resembling a big bang like event. In this case, an intermediate organism with only some of these components would have never existed.

## 13. MHC Locus Is Dynamic via ERVs

The MHC gene loci (I, II and III) encodes the TRC and Ig genes but also numerous other genes for processing and presentation of antigen, see [167]. These loci are the most polymorphic of all human genes. MHC class III is also the most gene dense (intron poor) region of human chromosome [168]. Many of the genes found within these loci are mammal and fish specific [168]. These genes have been linked together since the evolution of fish [169]. This raises the question regarding what was the source of most of these genes. The high plasticity of the MHC loci results from the action of many densely clustered retroelements [170]. The primate MHC loci are particularly dense with ERVs as they are in 10-fold greater abundance compared to other regions of the chromosomes [170,171]. High ERV MHC density is also seen in sharks, even though shark genomes do not host the same general high ERV level seen in primates [172]. MHC I appears to be ancestral to MHC II and their evolution has been by duplication and divergence of a core alpha block of genes [173,174]. ERVs are clearly involved in this duplication process, and with humans and chimp DNA, ERV9 can be used to categorize the types of duplication that has occurred [175]. The core MHC I ‘duplicon’ thus contains an ERV (HERV 16) [176–178]. MHC retroposon variation is especially found in introns of genes, resulting from HERVs and retrotransposon integration [179]. These retroposons are generating the diversity that also distinguish humans from chimp MHC loci. Interestingly, from a regulatory perspective, some of these MHC ERVs are retained in antisense orientation [180]. Thus there is little question that ERVs and retroposons provide the major variation in the MHC locus. Greater details regarding why viruses would be expected to have played such a role (from the perspective of identity systems) are presented in my previous review [148].

## 14. The Sudden Emergence of IFN Alpha and Gamma System

Along with the emergence of jawed vertebrates, we also see the emergence of both the interferon alpha and the interferon gamma system, crucial control systems that are both directly antiviral and initiate and maintain the adaptive immune response [181,182]. Comparative analysis of these two systems indicates that the interferon alpha system is more basal [152,183]. Although marine invertebrates have a virus associated dsRNA response with clear similarities to vertebrate interferon [184], this system differs notably from that of vertebrates. In jawed vertebrates, dsRNA longer than 30 bp can induce interferon which can also trigger many undesirable toxic side effects, such as apoptosis. Since dsRNA response systems were present before the emergence of vertebrates, it is simplest to propose that these systems were simply co-opted for use by the adaptive immune system. Yet the many tightly coordinated components of this response (such as NF-kappaB, [182]) still presents an evolutionary problem for attaining regulatory coherence. Invertebrates do not have the IFN alpha or gamma network, but various viruses clearly have or regulate essentially all of the genes involved.

## 15. T Cells Must Clonally Transform and Differentiate

As noted above, some major and coordinated changes were also needed in cell reprogramming (code editing) in order to create the tissue and cell types associated with the adaptive immune system. No single event would seem able to accomplish such a complex process. Yet, host cell transformation along with reprogramming differentiation is precisely the type of change viruses are evolved to perform. Indeed, HHV-6 for example can integrate [185], replicate nonlytically in dendritic cells and be required for efficient transmission and productive infection when cocultured with stimulated CD4 T cells (needing cell-cell contact) [186]. Similarly, CMV induces CD4+ and CD8+ cells to differentiate and over proliferate [187], resulting in T cell memory inflation [188]. EBV integration is distributed in chromosomes, providing potential regulatory links [189]. All these viral abilities would also have been important for the normal function of adaptive immunity. As genetic parasites viruses must be compatible with existing host code, but also extend it with new viral derived code to ensure virus maintenance or reproduction (and preclude competition). Indeed, both retroviruses and large DNA viruses of vertebrates and invertebrates are known to infect, transform and alter differentiation of immune cells or their stem cell precursors. Plus, gene sets within viruses would necessarily be under pre-coordinated viral regulatory control to operate with some coherence. Thus virus colonization provides a natural solution for reprogramming host cell fates. This reasoning was also previously developed to much greater detail [148].

## 16. Conclusions: Ongoing Viral Solutions to Adaptive and Other Immunity

We can find no extant organism that displays the gradual acquisition of the various components of the adaptive immune system. The complex coordinated regulation, along with complex coordinated dense gene sets involved do not exist outside of the jawed vertebrates. Yet, examples of most of these coordinated processes and genes can be found in viruses of various prokaryotes and eukaryotes. For example, viruses using various types of phase variation for receptor genes and addiction modules for self destruction are well known, see [148]. Thus a viral origin of such a linked process seems more plausible then a stepwise acquisition requiring reassignment of function. Viruses are known to encode and manipulate almost all aspects of the interferon network, see [190]. This is especially true for large persisting DNA viruses of the herpes family. The large DNA viruses of vertebrates are master modulators of MHC mediated immunity and persistence [190,191]. These herpesviruses show synchronous virus host evolution [192]. Some members, such as CMV, encode G coupled proteins and chemokines [193]. However such MCMV genes are usually assigned to evolve from host genes by gene piracy [194]. Thus, the similarities that viruses show to host interferon (or MHC) genes are broadly accepted to be due to viral piracy of host genes. In my judgment, such views are based on an incorrect assumption that any similarity between virus and host genes will necessarily support the idea of viral piracy of host. Yet many of these same viral genes are clearly unlike and often much simpler then any host version and are highly conserved in viral lineages [195]. Along these lines, I have argued vigorously that the converse situation is more likely true; that viruses originate these coordinated and dense gene sets associated with antiviral responses and have provided them to the host via colonization (a big bang like horizontal transfer). For example, CMV can completely suppress CTL mediated lysis [196]. This is a complex function that would also be essential for the origin of adaptive immunity itself in order to control the potential self destructive capacity of the system. CMV is a member of the herpesvirus family with ancient evolutionary links to phage. But unlike phage, the herpesviruses generally evolve via action of retrovirus and are intron poor. This contrasts with other DNA viruses including phycodnaviruses of algae (distant relative of herpes). Indeed some herpesvirus members can integrate fully functional retrovirus which results in an enhanced cooperating phenotype [197,198]. Clearly such a mixed virus must have attained regulatory coherence, yet it also has acquired the capacity for wide scale and distributed host integration with related regulatory elements. Thus virus evolution indeed can be mixed and complex. No single lineage of virus appears capable of providing all the complex changes needed to originate adaptive immunity. Complicated and cooperating mixtures of virus, however, do seem do able to provide these functions. Others have also previously considered whether viruses might have been involved in the origin of the adaptive immune system [199]. However, this was a much more narrow idea limited to the source of the RAG recombination system.

The acquisition of regulatory complexity, especially involving networks, appears to identify other key problems for recent human evolution. The great leaps forward or major evolutionary transitions associated with the emergence of humans (such as a large social brain) all seem to require coordinated regulatory changes, not simply new genes [200–202]. Thus the recent changes in the human genome are mostly found in non-coding DNA [203]. Here, HERVs and their LTRs appear to have had major effects on promoter or intron function in a process that appears to involve RNA interference and other regulatory mechanisms [204,205]. Some suggest a direct effect of LTRs on human promoters is small [206] although others claim human LTR promoter use is significant [207,208]. Few HERVs that are actively expressed in humans have open reading frames however [209]. Thus HERV/LTR effects are mostly RNA regulatory with an inherent potential to coordinate, even if sometimes the effects are deleterious [210]. With respect to immune system, here too it looks like HERV mediated changes predominate. Thus in spite of close overall conservation of human and chimpanzee ORFs, the dispersed indel differences in the MHC locus are considerable which results in species specific difference in retro and other viral sensitivities [211]. For example, the major MHC II polymorphism between human and chimpanzee via intronic sense HERVK31 and antisense for other ERVs is associated with lentivirus susceptibility [179]. Interestingly, EBV mediated introduction of HERV-K into primate cell lines has been reported suggesting virus cooperation [212]. Thus viruses are modifying antiviral systems in recent human evolution.

My objective for this review was to bring to the attention of readers the general involvement of viruses in antiviral systems. We have examined restriction-modification, CRISPERS, RNAi, and the adaptive immune system all from this perspective. In all cases, complex coordinated changes mediated by viruses can be observed. Essentially all antiviral systems appear to have some virus derived components. Perhaps because viruses can provide complex solutions, oppose virus competition and are able to inherently edit and extend host code they may be well suited for this general role.
